# Myasthenic congenital myopathy from recessive mutations at a single residue in Na_V_1.4

**DOI:** 10.1212/WNL.0000000000007185

**Published:** 2019-03-26

**Authors:** Nathaniel Elia, Johanna Palmio, Marisol Sampedro Castañeda, Perry B. Shieh, Marbella Quinonez, Tiina Suominen, Michael G. Hanna, Roope Männikkö, Bjarne Udd, Stephen C. Cannon

**Affiliations:** From the Departments of Physiology (N.E., M.Q., S.C.C.) and Neurology (P.B.S.), David Geffen School of Medicine at UCLA; Molecular and Cellular Integrative Physiology Program at UCLA (N.E., S.C.C.), Los Angeles, CA; Tampere Neuromuscular Center (J.P., T.S., B.U.), Tampere University and University Hospital, Finland; MRC Centre for Neuromuscular Diseases (M.S.C., M.G.H., R.M.), Department of Neuromuscular Disease, UCL Institute of Neurology, London, UK; Folkhälsan Genetic Institute (B.U.), Helsinki; and Neurology Department (B.U.), Vasa Central Hospital, Finland.

## Abstract

**Objective:**

To identify the genetic and physiologic basis for recessive myasthenic congenital myopathy in 2 families, suggestive of a channelopathy involving the sodium channel gene, *SCN4A*.

**Methods:**

A combination of whole exome sequencing and targeted mutation analysis, followed by voltage-clamp studies of mutant sodium channels expressed in fibroblasts (HEK cells) and *Xenopus* oocytes.

**Results:**

Missense mutations of the same residue in the skeletal muscle sodium channel, R1460 of Na_V_1.4, were identified in a family and a single patient of Finnish origin (p.R1460Q) and a proband in the United States (p.R1460W). Congenital hypotonia, breathing difficulties, bulbar weakness, and fatigability had recessive inheritance (homozygous p.R1460W or compound heterozygous p.R1460Q and p.R1059X), whereas carriers were either asymptomatic (p.R1460W) or had myotonia (p.R1460Q). Sodium currents conducted by mutant channels showed unusual mixed defects with both loss-of-function (reduced amplitude, hyperpolarized shift of inactivation) and gain-of-function (slower entry and faster recovery from inactivation) changes.

**Conclusions:**

Novel mutations in families with myasthenic congenital myopathy have been identified at p.R1460 of the sodium channel. Recessive inheritance, with experimentally established loss-of-function, is a consistent feature of sodium channel based myasthenia, whereas the mixed gain of function for p.R1460 may also cause susceptibility to myotonia.

Several allelic disorders of skeletal muscle are caused by mutations of *SCN4A* that encodes the pore-forming α subunit of the voltage-gated sodium channel (Na_V_1.4).^1^ Missense mutations with gain-of-function changes (GOF; too much inward Na^+^ current) are found in hyperkalemic periodic paralysis (HyperPP), paramyotonia congenita, and several variants of sodium channel myotonia.^2^ Leaky channels resulting from mutations of arginine residues in the voltage sensor domain cause hypokalemic periodic paralysis (HypoPP) type 2.^3,4^ These traits are all dominantly inherited.

Loss-of-function (LOF) mutations of *SCN4A* are encountered far less frequently and are associated with recessively inherited phenotypes. A congenital myasthenic syndrome with ptosis, bulbar weakness, respiratory difficulties, and prolonged episodes of weakness more typical for periodic paralysis has been associated with missense mutations of *SCN4A* that cause a LOF by markedly enhancing channel inactivation.^5–7^ More recently, congenital myopathy with neonatal hypotonia has been reported in patients with null mutations in *SCN4A*.^8^ A homozygous null is embryonic lethal, while compound heterozygous mutations (null allele plus an LOF allele) result in congenital myopathy with survival to adulthood. Remarkably, family members with a single *SCN4A* null allele are healthy.

In this report, we describe the molecular and clinical consequences of 2 additional LOF mutations, both of which are at residue p.1460. The index cases presented with congenital hypotonia, respiratory difficulties, and delayed motor milestones plus fatigue and were found to have biallelic mutations, as either p.R1460Q plus p.R1059X or homozygous p.R1460W. Expression studies of the p.R1460 mutant channels also revealed GOF changes that account for the myotonia in some carriers of p.R1460Q. Moreover, the phenotype for some carriers of the p.R1460Q mutation in the primary Finnish family was complicated by the independent cosegregation of a known *CLCN1* mutation p.R894X associated with recessive myotonia congenita.

## Methods

### Clinical examination

**Figure 1:**
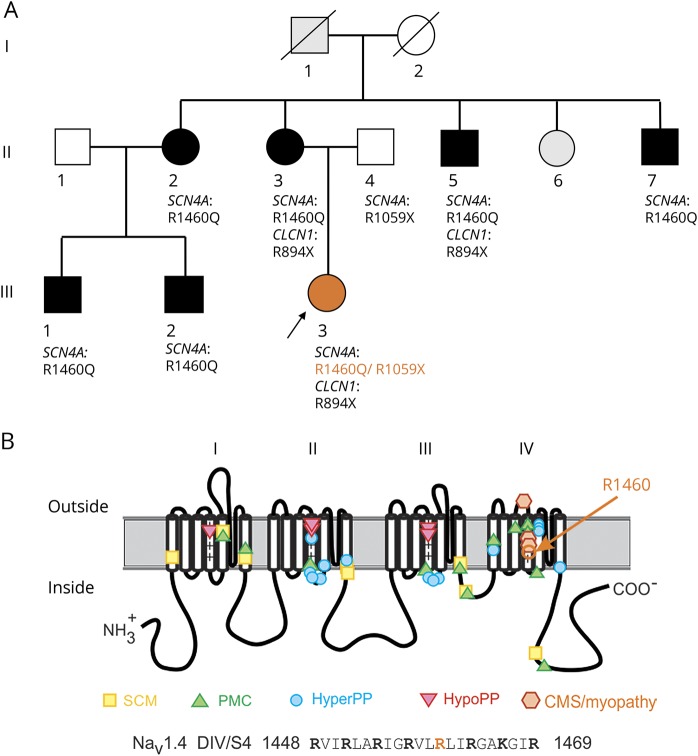
Sodium channel mutations (A) Segregation of clinical phenotype and genotype among 7 carriers of p.R1460Q in family 1 from Finland. (B) Location of p.R1460 in the pore-forming subunit (Na_V_1.4) along with established sites for sodium channelopathies of skeletal muscle. CMS = congenital myasthenic syndrome; HyperPP = hyperkalemic periodic paralysis; HypoPP = hypokalemic periodic paralysis; PAM = paramyotonia congenita; SCM = sodium channel myotonia.

The proband (III-3) and 6 of her relatives were examined in a Finnish family (F1, figure 1A). In addition, one of her aunts (II-6) and her maternal grandfather (I-1) had similar symptoms (larynx spasms) but were not available for further studies. The patients underwent neurologic examination, EMG, and DNA extraction. Further, a single unrelated Finnish patient (P2) with myotonia was similarly examined. Muscle histology was available for 2 patients and muscle MRI for 1 patient. The proband from family 2 and her parents were examined neurologically and whole blood was collected for DNA analysis.

### Clinical electrophysiology

Standard neurography and EMG investigation was performed in 9 patients with p.R1460Q mutation. Compound muscle action potential (CMAP) exercise test was carried out in 3 patients. A Fournier protocol was used with short (10–12 seconds) and long (5 minutes) exercise test.^9,10^ CMAPs were evoked by supramaximal nerve stimulation. The proband of family 1 also underwent repetitive nerve stimulation at 30 Hz and single-fiber jitter examinations. The proband of family 2 was studied by needle EMG and repetitive nerve stimulation at 3 Hz and 50 Hz. Because cooperation was limited in a 6-year-old patient, she did not complete a CMAP exercise test.

### Molecular genetics

The DNA of the proband in family 1 was studied by whole exome sequencing. The DNA sample was used for enrichment of a sequencing library by NimbleGen SeqCap EZ Human Exome v2.0 (Roche, Basel, Switzerland). The captured library was sequenced using an Illumina (San Diego, CA) HiSeq 2000 Sequencer. Sequence reads were aligned to the human reference genome (UCSC hg19) using the Burrows-Wheeler Aligner.^11^ Variant calling was made with Genome Analysis Toolkit.^12^ The data were visualized with the Integrative Genomics Viewer (Broad Institute, Cambridge, MA). DNA capture, enrichment, next-generation sequencing, and data analysis were performed at the Institute for Molecular Medicine Finland. From the sequencing data we selected to further analyze all the exons and exon–intron borders of the following genes: *MTM1*, *RYR1*, *DNM2*, *SEPN1*, and *SCN4A*. For the other members of family 1, the sequencing of relevant exons of *SCN4A* and *CLCN1* genes was performed using traditional Sanger sequencing (table). For some of the patients, 3 common Finnish mutations in the *CLCN1* gene (c.1238T>G p.F413C, c.1592C>T p.A531V, and c.2680C>T p.R894X) were studied by targeted mutation detection using TaqMan Sequence Detection System (ABI Prism 7000, Applied Biosystems, Foster City, CA). TaqMan Sequence Detection System is based on fluorescent oligonucleotide probes for both mutation and normal sequences. The studied fragments were amplified by PCR using forward and reverse primers. Sequences of the primers and probes are available upon request. DNA of patient P2 was sequenced using MYOcap targeted gene panel.^13^ Whole exome sequencing was performed for the proband in family 2 and her parents using the Agilent (Santa Clara, CA) SureSelect capture kit and an Illumina HiSeq2500 next-generation sequencer. Analysis of variants was performed by the UCLA Clinical Genomics Center.

**Table:**
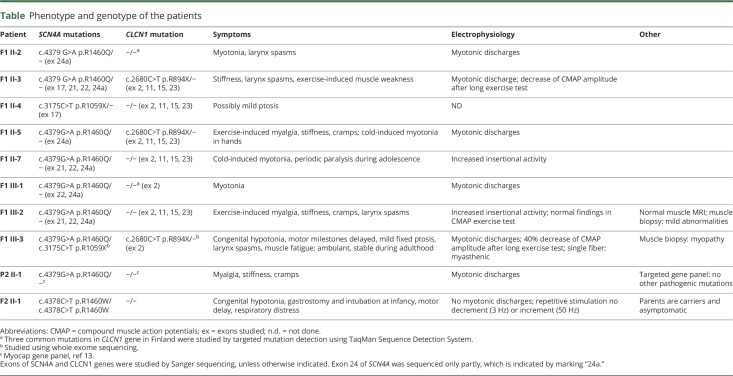
Phenotype and genotype of the patients

### Functional assessment of mutant sodium channels

**Figure 2:**
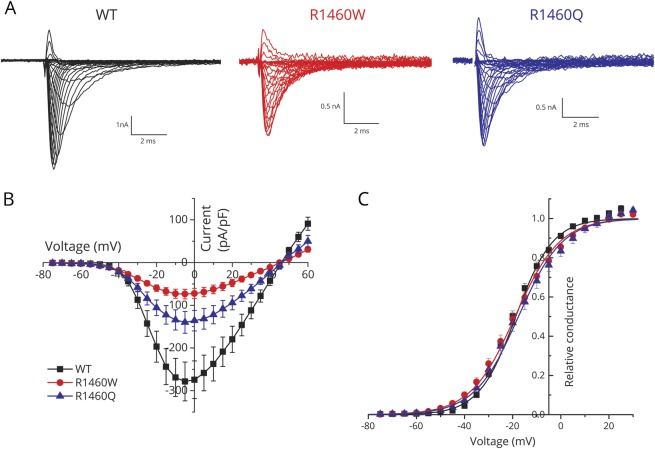
Sodium current density was reduced for cells transfected with R1460 mutant constructs, but the voltage dependence of activation was not altered (A) Traces show sodium currents elicited by depolarization from −120 mV to test potentials between −75 and +60 mV. Note the higher gain for the scale bars in the mutant channel traces. (B) Peak current density is shown as a function of test potential and displays a marked reduction in current amplitude for both mutants. (C) The voltage dependence of channel activation, as shown by relative conductance, was indistinguishable between the wild-type (WT) and mutant channels. Black squares, blue triangles, and red circles correspond to WT (n = 16), R1460Q (n = 12), and R1460W (n = 16), respectively.

Sodium channels were transiently expressed in fibroblasts (HEK cells) as previously described,^14^ except transfection was performed with lipofectamine. Cells expressed the human wild-type Na_V_1.4 pore-forming α subunit (WT), or mutant constructs encoding R1460Q or R1460W, plus the β_1_ accessory subunit. Sodium currents were recorded using the whole cell voltage-clamp configuration. The electrode contained the following, in mM: 100 CsF, 35 NaCl, 5 EGTA, 10 HEPES, pH to 7.3 with CsOH; the bath solution contained the following, in mM: 140 NaCl, 4 KCl, 2 CaCl_2_, 1 MgCl_2_, 2.5 glucose, 10 HEPES, pH to 7.3 with NaOH. After whole-cell access was achieved, cells were allowed to equilibrate for 5 to 10 minutes before recording. For the dataset to determine the voltage dependence of gating, cells with maximal peak Na^+^ currents <1 nA were excluded to minimize the contribution from endogenous Na^+^ currents (typically <0.1 nA), and those with peak current >5 nA were excluded to minimize series resistance errors. To reduce the selection bias for the current density measurements (figure 2), cells with peak Na^+^ currents from 0.5 nA to 10 nA were included. For the experiments to test for gating pore currents, channels were expressed in *Xenopus* oocytes, and currents were recorded using 2-electrode voltage clamp as previously described.^8^ The bath solution contained the following, in mM: 60 Na^+^-methansulfonate, 60 guanidine sulfate, 1.8 CaSO_4_, 10 HEPES, plus 1 μm tetrodotoxin to block sodium currents conducted by the central core.

The functional properties of WT and mutant sodium channels were compared quantitatively by obtaining parameter estimates to functions describing the voltage-dependent changes of the ionic currents. Channel activation was quantified by fitting the peak amplitude (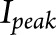) to a linear conductance (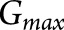) with a reversal potential (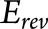) that was scaled with a Boltzmann function: 
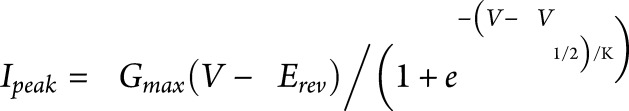
. The voltage-dependence for activation of the channel is characterized by 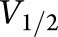, the voltage at which half the channels are activated, and 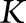, a steepness factor. The relative conductance (figure 2C) was calculated as 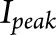 divided by 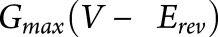. The time constant for entry to inactivation was estimated from a single exponential fit of the current decay (fast inactivation) or of the change in peak current after progressively longer conditioning pulses (closed-state inactivation). Steady-state fast inactivation was quantified by fitting the relative peak current at −10 mV after a 300 ms conditioning pulse (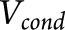) to a Boltzmann function 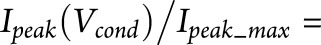 
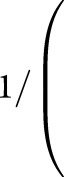
. For slow inactivation, a conditioning depolarization was used to induce inactivation, then a recovery pulse at −120 mV for 20 ms was applied to remove fast inactivation, and finally the available current (i.e., not slow-inactivated) was measured for a test pulse to −10 mV. For the steady-state voltage dependence, a plateau term (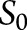) was included because slow inactivation does not reduce channel availability to 0 at strongly depolarized potentials. Estimated values for parameters are presented as mean ± SEM. Error bars in all graphs show ± SEM.

### Data availability statement

Photomicrographs of the muscle biopsy from the proband in family 1 are available upon reasonable request. The expression plasmids for the functional studies of mutant sodium channels are available upon request from the corresponding author.

### Standard protocol approvals, registrations, and patient consents

Informed written consent for genetic testing was obtained from patients or their guardians. Oocytes were harvested from adult female *Xenopus laevis* in accordance with the UK Animal (Scientific Procedures) Act 1986.

## Results

### Clinical presentation and assessment

#### Family 1

The proband (III-3), a 30-year-old woman, had congenital hypotonia that was severe at birth, causing difficulties in breathing and feeding, but resolved to milder nonprogressive muscle weakness and fatigue during the first years of life. The most severe symptoms resolved quickly in the first weeks of life; therefore, she did not need ventilator support or tube feeding. Motor milestones were delayed; independent sitting was achieved at 12 months but she started to walk at 18 months of age. From the start, she had severe weakness in the neck and facial muscles, mild dysphonia, and fixed ptosis bilaterally. Otherwise, muscle strength was 4/5 on the Medical Research Council Scale in all limbs. She had marked fatigability when climbing stairs; otherwise there was no large variability in her muscle strength. At age 30 years, she was ambulant, and could walk on toes but not on heels. Clinical myotonia was not detected.

Muscle biopsy showed myopathic changes with fiber size variation, increased internal nuclei, structural abnormalities, and necklace fibers. Some non-rimmed vacuolated fibers were seen. Several EMG studies were performed and one showed definite myotonic discharges. Single fiber test was myasthenic with increased jitter and the long-exercise test showed 40% decrease of CMAP amplitude. The proband had 2 mutations in the *SCN4A* gene: 1 missense p.R1460Q inherited from the mother and a nonsense p.R1059X inherited from the father. In addition, she had inherited a heterozygous *CLCN1* mutation p.R894X from her mother (figure 1). Whole exome sequencing did not reveal any other variants that might complicate the results. Genes associated with channelopathies (*SCN4A*, *CACNA1S*, *KCNJ2*, *CLCN1*) or congenital myasthenic syndrome and related disorders (*CHRNA1*, *CHRNB1*, *CHRND*, *CHRNE*, *RAPSN*, *CHAT*, *MUSK*, *COLQ*, *DOK7*, *AGRN*, *GFPT1*, *DPAGT1*, *LAMB2*, *CHRNG*, *PLEC*) were specifically checked.

The proband's mother, 3 siblings of the mother, and 2 cousins were identified with the p.R1460Q mutation. In addition, one unrelated patient (P2) carried the same mutation. They all had myotonic muscle symptoms and signs starting during young adulthood that either showed myotonic discharges or increased insertional activity on EMG and clinical myotonia, or related to muscle stiffness, exercise-induced myalgia, and cramps (table). Most of the p.R1460Q carrying patients in family 1 had larynx spasms that were evident in cold weather or at night. The *CLCN1* p.R894X mutation was also identified in the mother and one of her siblings. They had myotonic discharges on EMG but that was also seen in other siblings without *CLCN1* mutation. The father who carried the nonsense *SCN4A* mutation found in the proband possibly had mild fixed ptosis but was otherwise healthy.

#### Family 2

The proband was born with hypotonia. She exhibited poor feeding and respiratory insufficiency, requiring gastrostomy as well as intubation and mechanical ventilation. She was hospitalized for the first month of life. Motor milestones were attained including standing and walking, albeit delayed, and the parents reported day-to-day variability in strength and recurrent breathing difficulty. Around 6 years of age, she developed worsening motor function and intermittent respiratory distress, and was referred to the UCLA Neuromuscular Clinic for further evaluation. Her examination at that time demonstrated significant weakness of axial and appendicular muscles. She was not able to stand or support her head while in a seated positon. Reflexes were present, although mildly diminished. Repetitive stimulation of the median and accessory nerves at 3 Hz did not demonstrate a decrement in CMAP amplitude, and 50 Hz repetitive nerve stimulation of the median nerve did not elicit significant increment. No myotonic discharges were detected by needle EMG. Motor performance varied spontaneously, and on subsequent visits over the next 18 months she was able to stand and walk independently. She did not require bilevel positive airway pressure at night or other chronic ventilatory support. The patient's mother reported improvement of strength and breathing with inhaled albuterol, but the benefit was not sustained on oral therapy. A trial of pyridostigmine did not improve strength, but the family reported improved mobility when acetazolamide and albuterol were tried separately. The parents have no motor symptoms. An older sister reportedly had bulbar weakness, was never able to sit without support, and died at age 1 year from respiratory complications. The proband is homozygous for c.4378C>T p.R1460W in *SCN4A*, and both parents are asymptomatic carriers although there is no history of consanguinity in the family.

### Molecular results

The Na_V_1.4 variants in these families cause missense substitutions at the same amino acid, arginine 1,460, which is in the highly conserved S4 transmembrane segment in domain IV of the sodium channel (figure 1B). The allele frequency for p.R1460W in the gnomAD database is 6 per 276,796, and the p.R1460Q allele has not been reported. The S4 segments in the voltage sensors contain positively charged basic amino acids (arginine or lysine) at every third residue,^15^ and R1460 is the fifth of 8 such residues in S4 of domain IV. Although mutation at R1460 in Na_V_1.4 has not previously been reported in neuromuscular disorders, all 3 *SCN4A* mutations associated with a congenital myasthenic syndrome, and for which myopathy with fluctuating weakness is a prominent feature, are missense mutations of the voltage sensor in domain IV, and 2 are at other arginines in the S4 segment (p.R1454W and p.R1457H).^5–7^ All 3 previously reported mutations are also unusual because they cause LOF changes for the sodium channel and recessive inheritance of the syndrome, whereas GOF changes are found in *SCN4A* mutations associated with myotonia, paramyotonia, or HyperPP.^1^ We therefore tested the functional consequences of p.R1460Q and p.R1460W by recording sodium currents for channels heterologously expressed in fibroblasts or *Xenopus* oocytes.

### Functional characterization of R1460 mutant sodium channels

#### R1460 mutant sodium channels were expressed in the plasma membrane, but current density was reduced

Sodium currents were detected in HEK cells transfected with WT as well as R1460W and R1460Q mutant channels (figure 2). Peak current amplitudes were smaller, however, for cells expressing mutant channels. The peak current density (pA/pF to normalize for variations in cell size, figure 2B) for cells expressing mutant channels was about 30%–50% that of WT. At a test potential of – 5 mV, where the sodium current is largest, the amplitude was statistically smaller for each mutant compared to WT (*p* < 0.01, analysis of variance) but the difference between the 2 mutants was not distinguishable (R1460W −73.8 ± 11 pA/pF [n = 9], R1460Q −140 ± 26 pA/pF [n = 7], WT −278 ± 46 pA/pF [n = 12]).

### Channel activation was not altered by R1460Q or R1460W

The voltage dependence of sodium channel activation is shown in figure 1C, where the relative conductance is plotted as a function of the test potential (see Methods). The data are completely overlapping between WT and R1460 mutant channels. Quantitatively, there was no detectable difference in the voltage midpoint of activation with V_1/2_ of WT, R1460Q, and R1460W being −18.7 ± 1.1 mV (n = 16), −17.6 ± 1.7 mV (n = 12), and −19.0 ± 1.4 mV (n = 16), respectively. In addition, the steepness of the voltage dependence was similar for all 3: WT, R1460Q, and R1460W being 7.7 ± 0.22 mV, 9.1 ± 0.34 mV, and 9.2 ± 0.23 mV, respectively.

#### Fast inactivation had mixed, LOF, and GOF defects for R1460 mutant channels

**Figure 3:**
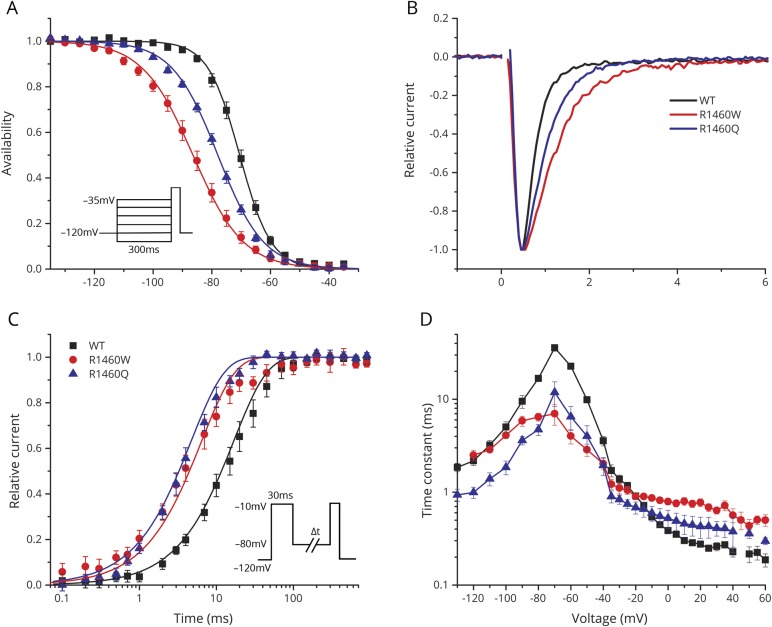
Fast inactivation of R1460 mutant channels had both gain-of-function and loss-of-function defects (A) The steady-state voltage dependence of fast inactivation was shifted leftward (hyperpolarized) for both R1460 mutant channels relative to wild-type (WT). Inset shows the voltage protocol used to measure inactivation produced by a 300 ms conditioning pulse at various potentials. (B) The rate of inactivation was slower for R1460 mutant channels at depolarized potentials, as shown by the superposition of amplitude normalized currents elicited at 10 mV. (C) Recovery from fast inactivation was faster for both R1460Q and R1460W, compared to WT channels (tau recovery of 4.7 ± 0.6 ms, *p* < 0.0001 [n = 5]; 6.4 ± 0.6 ms, *p* < 0.0001 [n = 7]; 16.8 ± 0.4 ms [n = 4], respectively). Data show the time course for the recovery of peak current amplitude at a holding potential of −80 mV, after channels were inactivated with a conditioning pulse of 30 ms at −10 mV (inset). (D) Plot summarizing the voltage-dependent kinetics for entry to or recovery from fast inactivation. Three separate protocols were used to measure inactivation kinetics, over the entire voltage range (see text, Results). Overall, the changes in steady-state fast inactivation (A) are loss of function, whereas the slower entry and more rapid recovery from fast inactivation are gain-of-function changes.

Sodium channels undergo 2 forms of inactivation; fast inactivation on a time scale of milliseconds, which contributes to termination of the action potential and limits repetitive firing, and slow inactivation over a time course of tens of seconds to minutes. To test the steady-state voltage dependence of fast invitation (also referred to as availability), we measured the peak current elicited at −10 mV, after a 300 ms conditioning pulse to potentials over a range from −130 to −40 mV (figure 3A). Depolarization promotes inactivation (reduces availability), and the voltage dependence was markedly left-shifted for R1460 mutant channels (V_1/2_ value for R1460Q −78.1 ± 0.67 mV, n = 10; R1460W −86.1 ± 1.5 mV, n = 12; and WT −70.5 ± 0.91 mV, n = 15). In addition, the steepness of the voltage dependence of steady-state invitation was reduced for R1460 mutant channels (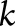 value for WT 5.5 ± 0.88 mV, R1460Q 8.6 ± 0.2 mV, R1460W 7.7 ± 0.2 mV). Combined, these changes in the voltage dependence of steady-state fast inactivation produce a substantial LOF, as can be seen by the reduced availability (figure 3A) of mutant channels (R1460Q = 0.7, R1460W = 0.5, compared to WT = 0.9) at the resting potential of −85 mV for skeletal muscle.

The voltage-dependent kinetics of fast inactivation were assessed for both entry to and recovery from inactivation. The entry rate was slower for R1460 mutant channels, which can be observed directly as a prolonged decay in a superposition of sodium currents elicited by a step depolarization to 10 mV (figure 3B). This slower entry rate was observed over a range of test potentials (−20 to +60 mV), as shown by the larger amplitude time constant of the current decay (figure 3D). Recovery from fast inactivation was recorded using a 2-pulse protocol. Channels were fast inactivated using a 30 ms conditioning pulse to −10 mV, then after a variable duration recovery at a hyperpolarized potential (−80 to −130 mV), a test pulse to −10 mV was delivered to determine the relative amplitude of the peak sodium current (figure 3C, inset). The time course of recovery at −80 mV is shown in figure 3C, and demonstrates the faster recovery (left shift) for R1460 mutant channels. A faster recovery rate (smaller time constant) of R1460 mutant channels was observed over a potential range from −50 to −130 mV (figure 3D). Finally, the kinetics of closed-state fast inactivation (figure 3D, −40 to −70mV) was measured using a 2-pulse protocol with a 30 ms conditioning pulse to induce inactivation, followed by a test pulse at −10 mV to measure the relative current. The altered entry and recovery kinetics for fast inactivation of R1460 mutants both contribute to a GOF change, in other words more inward sodium current. In the physiologic context of an action potential, mutant channels are predicted to inactivate more slowly at the peak of depolarization and then recover more quickly when the membrane potential returns to its resting value (−85 mV).

**Figure 4:**
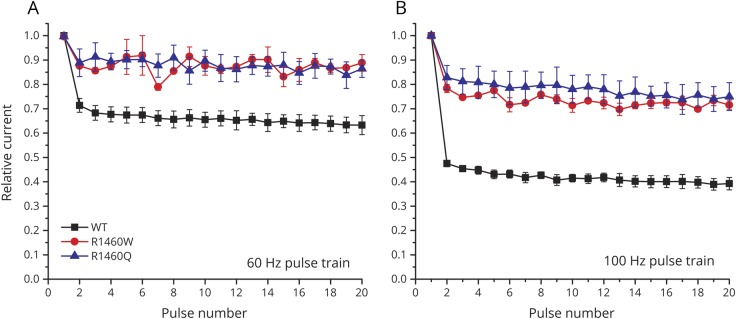
The use-dependent reduction in sodium current peak amplitude was less pronounced in R1460 mutants than in wild-type (WT) channels Data show relative peak sodium current elicited by 2 ms depolarizing pulses to 10 mV applied at 60 Hz (A) or 100 Hz (B), from a holding potential of −80 mV.

The impact of the altered kinetics of fast inactivation can be observed directly by measuring the peak amplitudes of sodium currents elicited by a train of brief depolarizing pulses. Normally, sodium channels do not have time to fully recover in the interval between action potentials with high-frequency discharges. We simulated this scenario by measuring sodium channel availability as the relative peak current elicited by a 2 ms depolarization to 10 mV, with an intervening recovery interval at −80 mV, over a range of pulse frequencies (figure 4). Use-dependent inactivation was pronounced for WT channels (e.g., 30% decrease at 60 Hz and 50% decrease at 100 Hz), but was substantially less for R1460 mutant channels. These data demonstrate a GOF for R1460 mutant channels that would manifest, for example, as myotonic bursts.

### Slow inactivation was unaffected for both R1460Q and R1460W mutant channels

**Figure 5:**
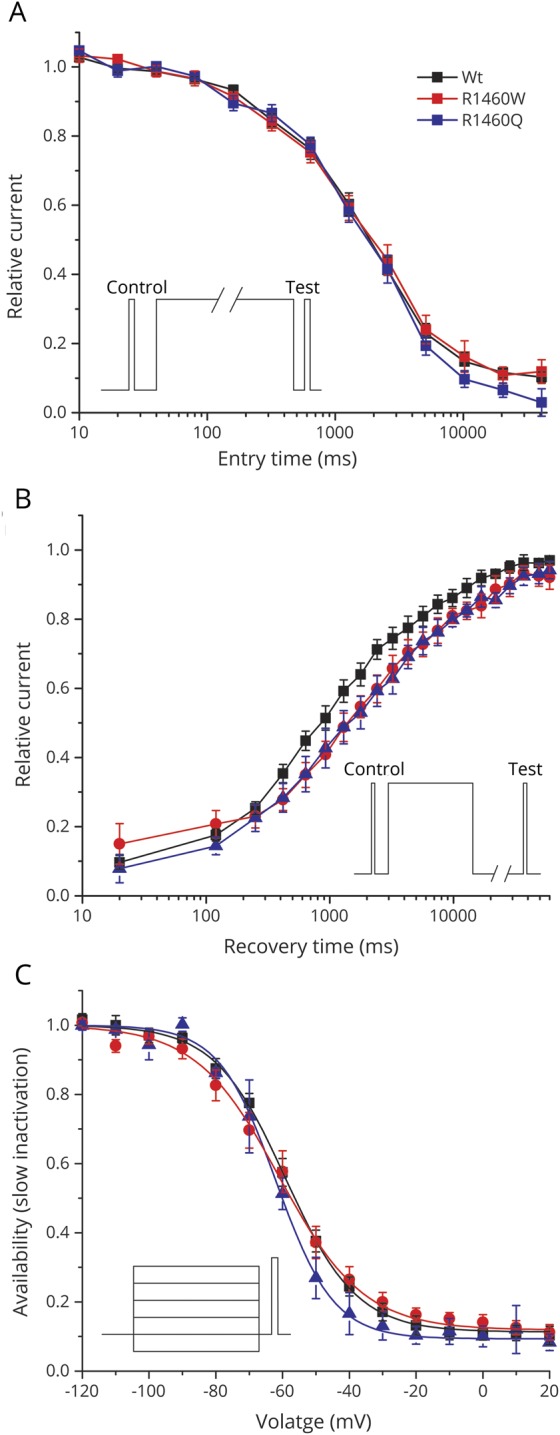
Slow inactivation was not altered by either R1460W or R1460Q (A) Onset of slow inactivation showed identical kinetics between wild-type (WT) and mutant channels. Inset shows the voltage protocol to characterize the onset of slow inactivation by stepping to −10 mV for a variable duration (entry time), and then measuring the decline in relative loss current that fails to recover within 20 ms at −120 mV. (B) The rate of recovery from slow inactivation was comparable between WT and R1460 mutant channels. Channels were slow inactivated by a 30-second step to −10 mV (inset), and the data show recovery as the relative increase in current after repolarizing to −80 mV for a variable duration (recovery time). (C) The voltage dependence of steady-state slow inactivation was indistinguishable between WT and R1460 mutant channels. Thirty-second conditioning pulses to various conditioning potentials (inset) were used to determine the voltage dependence of inactivation. Smooth curves show best fits to the data with V_1/2_ of −59.1 ± 1.9 mV for WT, −60.9 ± 2.8 mV for R1460Q, and −63.4 ± 2.6 mV for R1460W. The number of cells for the mean values shown in the data was 5–8 for WT and both R1460 mutant channels.

Prolonged depolarizations lasting seconds or sustained bursts of discharges both cause sodium channels to become slow inactivated, and derangements of slow inactivation are known to cause susceptibility to periodic paralysis^16^ or congenital myasthenia.^6^ We characterized slow inactivation by using prolonged step depolarizations (up to 60 seconds) to induce slow inactivation, followed by a 20 ms recovery period at −120 mV to remove fast inactivation, and then applied a test pulse to −10 mV to detect the presence of slow inactivation as a reduced peak current. The time course of entry to slow inactivation at −10 mV was indistinguishable between WT and R1460 mutant channels (figure 5A). Similarly, the rate of recovery was comparable for WT and R1460 mutant channels at −100 mV (figure 5B). The small rightward shift represents about a 1.5-fold slower rate of recovery for R1460 mutants, although the fitted time constants were not statistically distinguishable from those of WT channels. The voltage dependence of steady-state slow inactivation, as determined by 30-second conditioning pulses, was identical for WT and R1460 mutant channels (figure 5C).

### R1460 mutant channels do not conduct a gating pore leakage current

**Figure 6:**
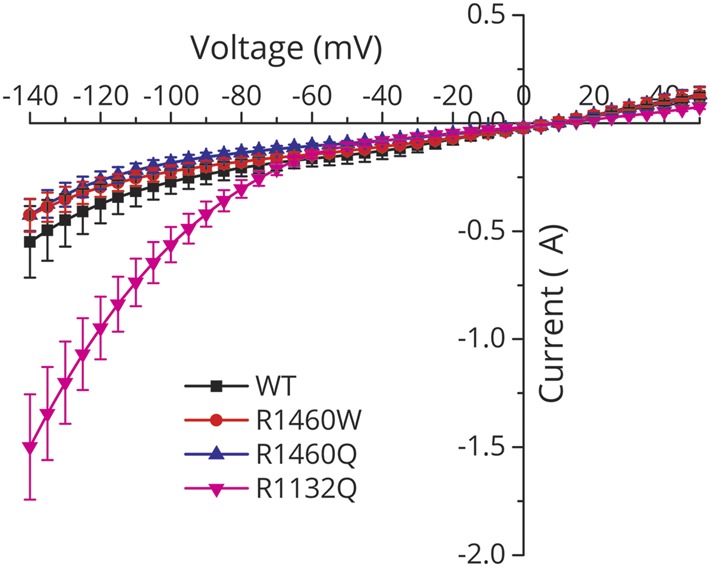
The R1460 mutations did not cause a gating pore leakage current The steady-state current (without subtraction of the nonspecific leak) recorded from oocytes expressing high levels of wild-type (WT) or R1460 mutant channels is plotted as a function of the membrane potential. Tetrodotoxin was added to block sodium currents through the main pore. The I–V relation was identical for WT and R1460 mutant channels, while the positive control for the p.R1132Q mutation in hypokalemic periodic paralysis showed a gating pore leakage current (large negative current for membrane potentials more negative than −80 mV). Data are from 6 to 8 oocytes per construct.

Missense mutations at arginine residues in the S4 segments of the voltage sensor domains of sodium or calcium channels account for almost all cases of HypoPP,^3^ and have a common functional defect, the gating pore leakage current, caused by a mutation-induced anomalous ion conduction pathway.^4^ Since R1460 is an arginine in the S4 segment of domain IV in Na_V_1.4 (figure 1B), we tested whether R1460Q or R1460W mutant channels have a detectable gating pore leakage current. Channels were studied in *Xenopus* oocytes to achieve high expression levels sufficient to observe gating pore currents when the main pore was blocked by tetrodotoxin. Currents recorded from oocytes expressing R1460Q or R1460W channels were no different from WT (figure 6), as compared to the positive control with the HypoPP mutation R1132Q, for which a large gating pore current was detected (inward current a negative potentials).

## Discussion

This report identifies a new residue in Na_V_1.4 (R1460) where missense mutations were identified in patients with recessive myasthenic congenital myopathy. The clinical manifestations overlap considerably with prior reports of a recessive congenital myasthenia syndrome plus periodic paralysis that is associated with LOF mutations in SCN4A.^5–7^ The consistent features are hypotonia at birth, respiratory distress, and feeding difficulties often requiring tube feeds. Motor function at infancy and early childhood is notable for delayed milestone attainments, fixed ptosis, myopathic weakness especially of the face and neck, episodes of hypoventilation, myalgia, and fatigability.

Electrophysiologic signs of myasthenia are variable. The first published report (p.S246L/p.V1442E) was notable for pronounced CMAP decrement during repetitive nerve stimulation (at 2 Hz after a 10 Hz load and at all frequencies of 10 Hz or higher).^5^ For the 2 subsequent single-case reports of *SCN4A*-associated congenital myasthenic syndrome (CMS), one had increased jitter^7^ (as observed for patient F1 III-3 herein) but neither had a CMAP decrement with repetitive stimulation. Conversely, in the series of 6 families with congenital myopathy associated with *SCN4A* recessive mutations, 1 of 4 available surviving patients had a 60% CMAP decrement with 10 Hz stimulation.^8^

A consistent pattern is emerging for the genotype–phenotype associations with LOF mutations in *SCN4A*. A single mutant allele, whether a partial loss or complete null, is asymptomatic, although a mouse model revealed a latent susceptibility to myasthenia.^17^ A biallelic deficit with homozygous LOF mutations leads to moderate congenital myopathy with fatigue and variable electrodiagnostic signs of myasthenia. Compound heterozygous mutations with a null plus a LOF defect cause moderate to severe congenital myopathy, including an increased risk of infant mortality. Finally, biallelic null mutations, whether from nonsense mutations or missense mutations with no detectable sodium current, are lethal in utero or at birth.^8^ In hindsight, this paradigm suggests the original report of *SCN4A*-associated CMS was indeed caused by a recessive compound heterozygote (p.S246L/p.V1442E).^5^ Because the fast-inactivation defect for p.V1442E was much greater (−33 mV shift) than for p.S246L (−7.3 mV), we initially considered p.S246L to possibly be a benign polymorphism. In retrospect, the substantial enhancement of slow inactivation for p.S246L (−20 mV shift) will reduce Na_V_1.4 availability at V_rest_ by ∼30%. Recognizing now that a single LOF allele is asymptomatic and that p.S246L does not appear in the gnomAD database strongly supports the notion that S246L is a functionally relevant LOF mutation.

The clinical phenotypes associated with mutations at p.R1460 described herein were more varied, which we attribute to the mixed LOF and GOF changes observed for both R1460Q and R1460W mutant channels. In the primary Finnish family, inheritance of myasthenic congenital myopathy was recessive (p.R1460Q/p.R1059X) but myotonia was a partially dominant trait. Latent myotonia was detected by needle EMG in p.R1460Q carriers, and some had symptomatic myotonia, especially with the coincident occurrence of an independently segregating mutation of the chloride channel gene *CLCN1* (p.R894X associated with recessive myotonia congenita). Combined GOF mutations in *SCN4A* and LOF mutations in *CLCN1* are known to synergistically aggravate the severity of myotonia.^18,19^ The LOF defects for R1460W channels were more severe than those of R1460Q (smaller amplitude, figure 2; greater left shift of inactivation, figure 3), which may explain why the parents shown to be p.R1460W carriers did not have myotonia. Unusual clinical phenotypes have also been reported for missense mutations of the second arginine in the voltage sensor of domain IV (p.R1451L and p.R1451C).^20,21^ In the heterozygous state, p.R1451L is associated with myotonia aggravated by cold and rare episodes of periodic paralysis typical for paramyotonia congenita, which is consistent with GOF defects manifest as slower entry and faster recovery from inactivation. An individual homozygous for p.R1451L had more frequent and severe episodes of weakness, one of which with serum K^+^ 2.8 mM was suggestive of HypoPP.^21^ Expression studies of R1451L did not reveal a gating pore leakage current—the canonical defect in HypoPP—but instead showed LOF changes (reduced peak current and enhanced inactivation) that may cause this unusual form of periodic paralysis in homozygous patients.^21^ Unlike the cases herein, however, the LOF aspects of the mixed channel defects for p.R1451L did not result in a CMS syndrome in the homozygous proband.

Our report is the third case of a homozygous recessive mutation in *SCN4A* found in CMS/congenital myopathy, and all 3 missense mutations are at arginine residues in the S4 segment of the domain IV voltage sensor (p.R1454W^6^, p.R1457H^7^, and p.R1460W). Expression studies for all 3 mutations show a similar pattern of LOF changes with aberrant enhancement of inactivation, consistent with the established structure–function behavior of voltage-gated sodium channels; the domain IV sensor is coupled to inactivation^22^ whereas domains I–III control activation. All 11 mutations of Na_V_1.4 that cause HypoPP type 2 are also missense substitutions of arginines in S4 segments within voltage sensors.^1,3^ These mutations all create anomalous “gating pore” leakage currents that are mechanistically implicated in episodic attacks of depolarization and weakness in HypoPP.^4^ The HypoPP mutations of S4 are in domains I–III, whereas a homologous mutation in domain IV (p.R1448C) causes paramyotonia congenita and does not create a gating pore leak.^23^ Scanning mutagenesis studies have shown that a missense substitution of any single arginine in S4/DIV is insufficient to create a gating pore leak,^24^ and we have confirmed herein that the p.R1460 mutations found in recessive congenital myopathy do not produce a leak (figure 6).

Further investigations are required to understand the mechanism for the fatigue in CMS/congenital myopathy caused by mutations in *SCN4A*. Prolonged episodes of weakness reminiscent of periodic paralysis are not likely to be caused by sustained fiber depolarization, as with attacks of HyperPP or HypoPP, because this clinical symptom occurs with CMS mutations that lack GOF changes (e.g., p.R1454W^6^). Fatigue in this recessive phenotype is more likely to be a consequence of impaired generation and conduction of action potentials caused by a marginal sodium current density that may be exacerbated by use-dependent trapping of sodium channels in an inactive state.

## Glossary

CMAP: compound muscle action potential
CMS: congenital myasthenic syndrome
GOF: gain-of-function
HyperPP: hyperkalemic periodic paralysis
HypoPP: hypokalemic periodic paralysis
LOF: loss-of-function
WT: wild-type
